# Novel *SPAST* deletion and reduced *DPY30* expression in a Spastic Paraplegia type 4 kindred

**DOI:** 10.1186/1471-2350-15-39

**Published:** 2014-04-01

**Authors:** Loretta Racis, Eugenia Storti, Maura Pugliatti, Virgilio Agnetti, Alessandra Tessa, Filippo M Santorelli

**Affiliations:** 1 Department of Clinical and Experimental Medicine, University of Sassari, Sassari, Italy; 2 Department of Biomedical Sciences, University of Sassari, Sassari, Italy; 3 IRCCS Stella Maris, via dei Giacinti 2, 56028 Pisa, Italy

**Keywords:** SPG4, *DPY30*, Genetic modifier, Deletion

## Abstract

**Background:**

The hereditary spastic paraplegias (HSPs) are pleiomorphic disorders of motor pathway and a large number of affected genes have been discovered. Yet, mutations in SPG4/*SPAST* represent the most frequent molecular etiology in autosomal dominant (AD) patients and sporadic cases. We describe a large, AD-HSP Sardinian family where 5 out of several living members harbored a novel deletion affecting also the 5′UTR of *SPAST* and resulting in reduced expression of *DPY30*, the gene located upstream *SPAST* in a head-to-head manner.

**Case presentation:**

A 54-year-old woman manifested leg stiffness at age 39 and required a cane to walk at age 50. Neurological examination disclosed mild spasticity and weakness in the legs, hyperreflexia in all limbs, and bilateral Babinski sign. She also complained of urinary urgency, but no additional neurological symptoms or signs were detected at examination. The clinical examination of 24 additional relatives disclosed three further affected individuals, two men and one woman. In the four symptomatic patients the initial manifestations were walking abnormalities and leg stiffness with a mean age at onset (SD) of 46.75 (5.44) years (range 39–51). The mean disease duration was 13.2 (13.4) years (range 6–35), and it correlated well with clinical severity (SPRS score) (*r* = 0.975, *p* = 0.005). One patient was confined to bed and displayed knee and ankle contractures, another case needed a cane to walk, and two individuals were able to walk without aids. Interestingly, a patient had also had a miscarriage during her first pregnancy.

Gene testing revealed an heterozygous deletion spanning from the 5′-UTR to intron 4 of *SPAST* in the affected individuals and in one clinically unaffected woman. In three affected patients, the deletion also determined low mRNA levels of *SPAST* and *DPY30*, a component of the Set1-like multiprotein histone methyltransferase complex located upstream, head-to-head with *SPAST.*

**Conclusion:**

Together with data described in a Japanese family, our findings seem to suggest that genes close to spastin might be candidates in modulating the clinical phenotype. This report endorses future research on the role of neighboring genes as potential players in SPG4 disease variability.

## Background

Heterogeneity is a key feature of the hereditary spastic paraplegias (HSPs). To date, autosomal, sex-linked, and cytoplasmic inheritance have been reported, an ample array of complicated phenotypes disclosed, more than 70 loci mapped, and roughly 50 disease genes cloned [1,2]. In common clinical practice, the gene-after-gene testing strategy allows definition of the molecular basis in about half of HSP cases, unless peculiar features emerge during examination or follow-up.

Mutations in *SPAST*/SPG4 encoding spastin represent the most common cause of autosomal dominant hereditary spastic paraplegias (AD-HSP) and also account for about 15% of sporadic cases [3,4]. In north-west Sardinia the relative frequency of HSP is higher than what is calculated in other Western European populations with an estimated crude prevalence of about 17.5/100,000 for AD-HSP [5]. As documented in several families and different populations [4,6], the SPG4 phenotype is usually pure and inter- and intra-familial variability of the clinical presentation are well established [7]. In some cases, single nucleotide polymorphisms in *SPAST* and variants in additional genes are invoked as modifiers of age at onset, disease course and severity [8,9].

We identified a novel *SPAST* mutation segregating in a Sardinian kindred (family IK).

## Case presentation

**Figure 1:**
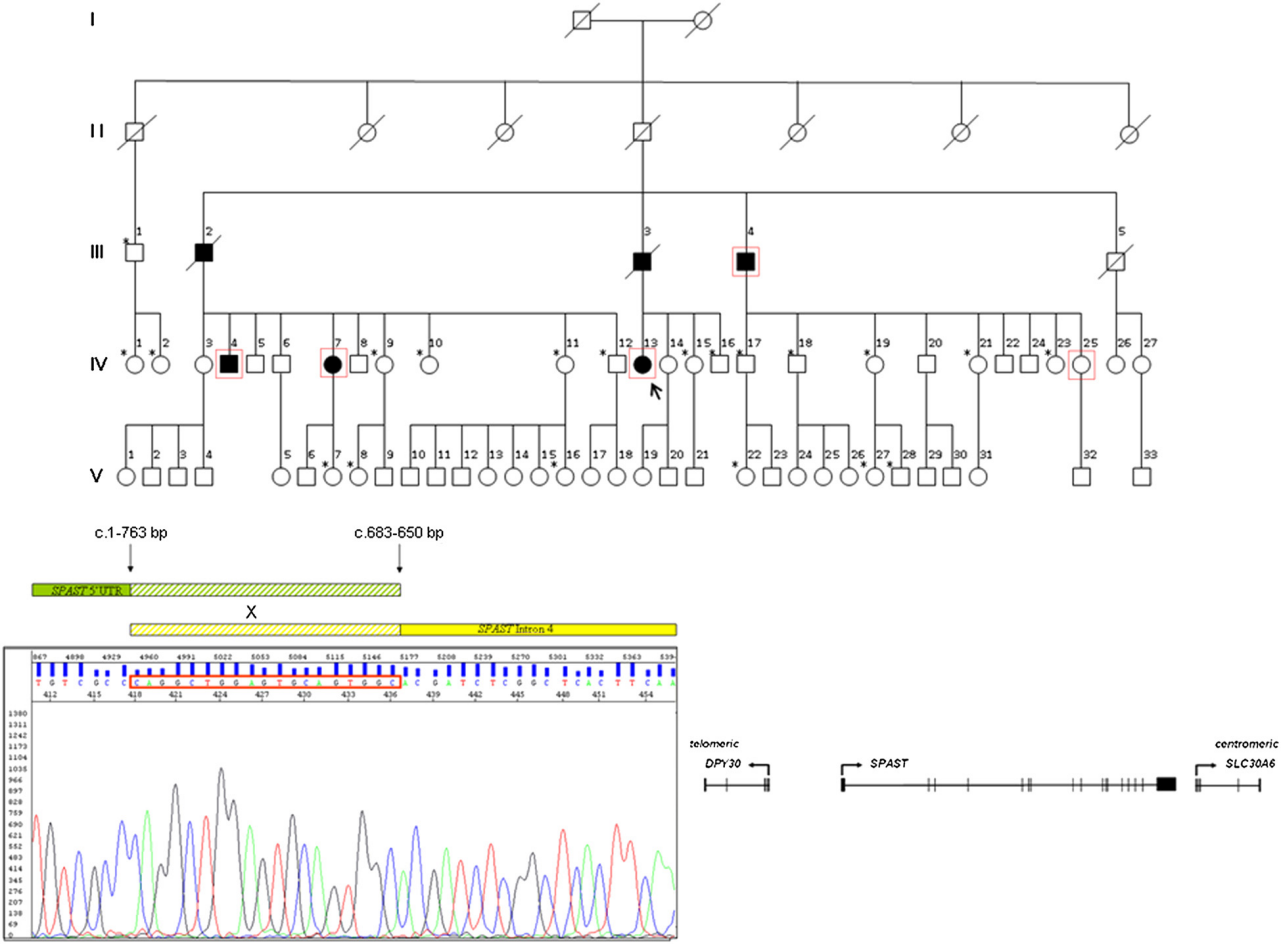
**(*Top*) Family pedigree.** The arrow indicates the index case. Squares, men; Circles, women. Black symbols indicate clinically affected individuals; empty symbols show clinically healthy relatives. Carriers of the novel mutation in SPG4/*SPAST* are indicated with a dotted frame. Star symbols indicate individuals who underwent DNA studies but then tested normal. (*Bottom*) Electropherogram flanking the novel mutation c.1-763_c.683-650del affecting the 5′UTR and exons 1–4 of *SPAST*, and the position of spastin neighboring genes.

The proband, a 54-year-old woman (IV-13) (Figure 1), manifested leg stiffness at age 39 and required a cane to walk at age 50. Neurological examination disclosed mild spasticity and weakness in the legs, enhanced deep tendon reflexes in all limbs, and bilateral Babinski sign. Case IV-13 also complained of urinary urgency, but no additional neurological symptoms or signs were detected at examination. The patient reported that her deceased father (III-03) and two uncles (III-02, III-04) had manifested similar gait abnormalities in adult age, although with different degree. Detailed clinical information and examinations were gathered from her living relatives.

## Materials and methods

Total genomic DNA was purified by peripheral blood with standard methodologies. Analysis of common AD genes associated with HSP used traditional Sanger sequencing and the BigDye 3.1 Chemistry, as reported [5]. Search for copy number variation and gene deletion/duplication adopted reported array-comparative genomic hybridization (aCGH) and multiple ligation-dependent probe amplication (MLPA) methodologies [10].

**Figure 2:**
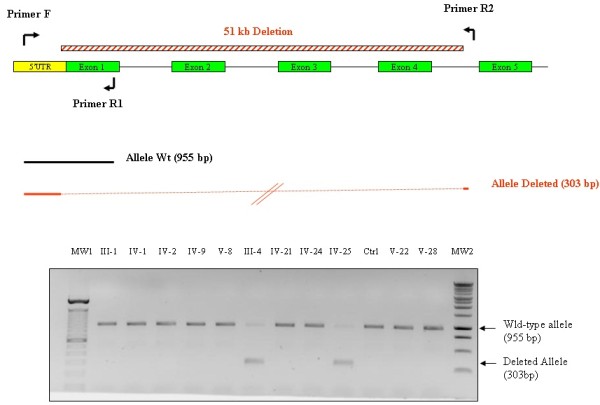
Rapid diagnostic genotyping of the multi-exon deletion detected in the IK family by a duplex-PCR method.

Twenty-four additional relatives were examined and sampled with written informed consent in family IK. Using oligonucleotide primers (5′-3′) SPASTPrimer F, SPASTPrimer R1 and SPASTPrimer R2 that were designed flanking the mutation deletion breakpoints (sequences available upon request), we PCR-amplified genomic DNA to generate a single 955-bp fragment in wild-type individuals. The presence of the heterozygous *SPAST* deletion produced an additional fragment of 303-bp (Figure 2).

To test the effects on mRNA expression of the mutation deletion in *SPAST*, total blood RNA was extracted using a micro-scale total RNA separator kit (Ambion INC., Austin, TX). For standard gene expression experiments, the mRNA transcript levels were determined by qPCR runs in an ABI7500Fast system (Applied Biosystems, Foster City, CA) using the TaqMan Universal PCR Protocol, and human *SPAST* (Hs00368084_m1, Applied Biosystems), *SLC30A6* (Hs01071782_m1), and *DPY30* (Hs00261491_m1) as probes. *GAPDH* (Hs99999905_m1, Applied Biosystems) was used for endogenous normalization, and expressions were determined using the comparative Ct method [11]. Values were normalized in reference to the average control value obtained from three age-matched normal control subjects. Statistical analyses used unpaired two-tailed Student-test (significance was set at p <0.05).

## Results

No point mutations were found in *SPAST* and analyses of other frequent AD-HSP etiologies (namely, SPG3A/*ATL1*, SPG31/*REEP1*, SPG10/*KIF5A*, SPG8/*KIAA0196*) were all normal in the proband. Combination of customized aCGH, MLPA analysis, and direct sequencing identified a novel heterozygous mutation (c.1-763_c.683-650del) spanning 51 kb, from the 5′-UTR (and upstream regulatory elements) to intron 4 of *SPAST*. The deletion mutation was not found in the NCBI genomic structural variations database (http://www.ncbi.nlm.nih.gov/dbvar/?term=human+SPAST) nor in polymorphic databases.

Using a rapid PCR-based method to quickly genotype individuals in the family, and rule out the mutation in 500 ethnically-matched control chromosomes, we identified the new mutation in a total of five individuals, including the yet asymptomatic IV-25 (Figure 1). In all, global disease severity was assessed using the Spastic Paraplegia Rating Scale (SPRS) [12]. Table 1 summarizes clinical data in carriers of the novel *SPAST* mutation deletion. In the four symptomatic patients the initial manifestations were walking abnormalities and leg stiffness with a mean age at onset (SD) of 46.75 (5.44) years (range 39–51). The mean disease duration was 13.2 (13.4) years (range 6–35). Pearson’s correlation coefficient indicated a positive correlation between disease duration and clinical severity (SPRS score) (*r* = 0.975, *p* = 0.005). One patient (III-04) was confined to bed and displayed knee and ankle contractures, another needed a cane to walk, and two individuals were able to walk without aids. Interestingly, subject IV-07 had also had a miscarriage during her first pregnancy. At this point, we cannot state if IV-25 will develop any clinical manifestation in the near future or if she is a true not penetrant carrier of the mutation, since her present age is slightly younger than the mean age of onset of motor disturbances in the family. Reduced or no penetrance has repeatedly been described in spastin-related HSP kindred [1,3], including cases from Sardinia [10].

**Table 1:** Clinical features in the five patients harboring the mutation in the IK family

	**III-04**	**IV-04**	**IV-07**	**IV-13**	**IV-25**
**Age/Sex**	86/M	53/M	60/F	54/F	42/F
**Age at Onset, yrs**	51	47	50	39	NA
**Disease duration, yrs**	35	6	10	15	0
**SPRS score**^**a**^	47	12	6	16	0
**SPRS item 13**^**b**^	4	1	1	1	0
**Spasticity score LL**^**c**^	4	2	1	2	0
**Muscle strenght score LL**^**d**^	0	5	4	4	5
**Hyperreflexia UL/LL**	Contracture	0/+ + +	0/+ +	+/+ + +	0/0
**Babinski sign**	+	+	Indifferent	+	Indifferent
**Decreased vibration sense**	na	No	No	No	No
**EMG/NCS**	na/na	Abnormal*/n	n/n	n/n	na/na
**Miscarriage/Pregnancy**			1/3	0/0	0/1

*M* man, *F* woman, *UL* Upper Limbs, *LL* Lower Limbs, *na* not available, *EMG* electromyography, *NCS* nerve conduction studies, *n* Normal; *slight impairment but no frank signs of denervation.

^a^The SPRS score ranges from 0 (normal) to 52 (severely affected) [12]; ^b^1 = Urinary urgency (difficulties to reach toilet in time); 4 = Permanent catheterization; ^c^The Modified Ashworth Scale of Muscle Spasticity score ranges from 0 (no increase in tone) to 4 (affected parts rigid in flexion or extension) [13]; ^d^The Medical Research Council Scale for Muscle Strength score ranges from 0 (no contraction) to 5 (normal strength) [14].

**Figure 3:**
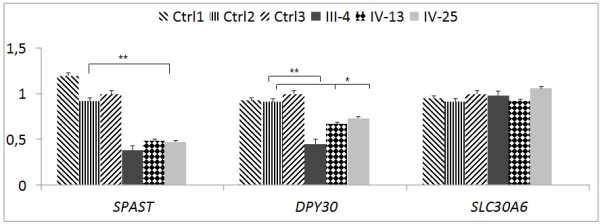
**Bar chart showing the relative expression of mRNA/*SPAST*, mRNA/*DPY30*, and mRNA/*SLC30A6 *in blood.** Data are presented from three patients in the IK family (samples refer to pedigree in Figure 1) with error bars representing the mean ± SD of three different determinations. Values are reported as the ratio to the internal control (GAPDH), and are also expressed in reference to the average control value from three age-matched normal subjects (Ctrls). **, p < 0.01; *, p < 0.05.

In three affected patients, we observed that the deletion also determined low mRNA levels of *SPAST* and *DPY30*, a component of the Set1-like multiprotein histone methyltransferase complex located upstream, head-to-head with *SPAST*[15] (Figure 3).

## Discussion

This report is the third description of a deletion characterizing the 5′-UTR of *SPAST*. Interestingly, the mutation also affected even more upstream sequences likely regulating *SPAST* and the neighbor *DPY30* gene. Previously, a deletion was found in a Japanese family with clinical features and disease duration highly similar to our cases [16]. Also, six men and four women in a further Japanese kindred harbored a 70 kb deletion involving exons 1 to 4 of *SPAST* and also exons 1 to 3 of *DPY30*[17]. Those patients had on average a teenage onset and a slowly progressive course leading to wheelchair in four, and use of a walking stick in one case. Other clinical features in that family were mild cognitive impairment and slight peripheral neuropathy. We believe that the presence of the spastin deletion might well explain a tendency towards less severe walking disability in family IK and in the Japanese kindred as described before in other families [3,9]. It is, however, intriguing that all affected Japanese women experienced miscarriages of unknown etiology similar to subject IV-07 in our kindred. Whether the shared feature of birth interruption relates to a similar defect in *DPY30* is still questionable.

*DPY30* is yet to be fully characterized but it seems to be essential for neural fate of embryonic cells, cell-cycling and cellular proliferation [15,18]. In nematodes, null mutations in dpy-30 cause XX-specific lethality and the gene is required for normal development of XO males [19]. As it orthologue, it can be speculated that human *DPY30* is also implicated in brain development and infertility [20] and when mutated might lead to miscarriages. The hypothetical function of the gene, however, cannot clearly explain how a low *DPY30*/mRNA expression (probably because of a position effect involving long-range gene regulatory elements) in the IK family with a milder phenotype agrees with the reduced mRNA levels (because of partial gene deletion) in the Japanese family with an earlier onset and apparently more severe neurological features [17]. We have no clear-cut explanation for this apparent “clinical riddle” other than raising the possibility of additional modifiers in the two families, maybe related to the different ethnic origin. Yet, alike the recent identification of a small deletion of *SLC30A6* — the gene flanking *SPAST* 3′UTR — in another Italian SPG4 family [10], this report endorses future research on the role of neighboring genes as potential players in SPG4 disease variability.

## Conclusion

We describe an AD-HSP Sardinian family where 5 out of several living members harbored a novel deletion affecting also the 5′UTR of *SPAST* and resulting in reduced expression of *DPY30*, the gene upstream *SPAST* in a head-to-head manner. If the presence of the spastin deletion might well explain a tendency towards less severe walking disability in our family, it is intriguing that a patient in our kindred experienced a miscarriage of unknown etiology similar to all affected women in a Japanese family harboring a *SPAST* and *DPY30* deletion. This report encourages future research on the role of neighboring genes as potential players in SPG4 disease variability.

## Consent

Written informed consent was obtained from the patients in the family for publication of this Case report.

## Competing interest

The authors declare that they have no competing interests.

## Authors’ contributions

LR was involved in the acquisition and analysis of clinical data. AT and ES carried out the molecular genetic studies and participated in drafting the manuscript. MP was involved in the collection of patients and in the acquisition and analysis of clinical data. VA participated in the coordination of the study and revised the draft critically. FMS and LR conceived the study, participated in its design and coordination and contributed to draft the manuscript. All authors read and approved the final manuscript.

## Pre-publication history

The pre-publication history for this paper can be accessed here:

http://www.biomedcentral.com/1471-2350/15/39/prepub
